# Candidate SNP markers of aggressiveness-related complications and comorbidities of genetic diseases are predicted by a significant change in the affinity of TATA-binding protein for human gene promoters

**DOI:** 10.1186/s12864-016-3353-3

**Published:** 2016-12-28

**Authors:** Irina V. Chadaeva, Mikhail P. Ponomarenko, Dmitry A. Rasskazov, Ekaterina B. Sharypova, Elena V. Kashina, Marina Yu Matveeva, Tatjana V. Arshinova, Petr M. Ponomarenko, Olga V. Arkova, Natalia P. Bondar, Ludmila K. Savinkova, Nikolay A. Kolchanov

**Affiliations:** 1 Institute of Cytology and Genetics, Siberian Branch of Russian Academy of Sciences, 10 Lavrentyev Avenue, Novosibirsk, 630090 Russia; 2 Novosibirsk State University, 2 Pirogova Street, Novosibirsk, 630090 Russia; 3 Children’s Hospital Los Angeles, 4640 Hollywood Boulevard, University of Southern California, Los Angeles, CA 90027 USA; 4 Vector-Best Inc, Koltsovo, Novosibirsk Region 630559 Russia

**Keywords:** Aggressiveness, Gene, Promoter, TATA-binding protein, Single nucleotide polymorphism, Candidate SNP marker, Keyword-based search, Prediction *in silico*

## Abstract

**Background:**

Aggressiveness in humans is a hereditary behavioral trait that mobilizes all systems of the body—first of all, the nervous and endocrine systems, and then the respiratory, vascular, muscular, and others—e.g., for the defense of oneself, children, family, shelter, territory, and other possessions as well as personal interests. The level of aggressiveness of a person determines many other characteristics of quality of life and lifespan, acting as a stress factor. Aggressive behavior depends on many parameters such as age, gender, diseases and treatment, diet, and environmental conditions. Among them, genetic factors are believed to be the main parameters that are well-studied at the factual level, but in actuality, genome-wide studies of aggressive behavior appeared relatively recently. One of the biggest projects of the modern science—1000 Genomes—involves identification of single nucleotide polymorphisms (SNPs), i.e., differences of individual genomes from the reference genome. SNPs can be associated with hereditary diseases, their complications, comorbidities, and responses to stress or a drug. Clinical comparisons between cohorts of patients and healthy volunteers (as a control) allow for identifying SNPs whose allele frequencies significantly separate them from one another as markers of the above conditions. Computer-based preliminary analysis of millions of SNPs detected by the 1000 Genomes project can accelerate clinical search for SNP markers due to preliminary whole-genome search for the most meaningful candidate SNP markers and discarding of neutral and poorly substantiated SNPs.

**Results:**

Here, we combine two computer-based search methods for SNPs (that alter gene expression) {i} Web service SNP_TATA_Comparator (DNA sequence analysis) and {ii} PubMed-based manual search for articles on aggressiveness using heuristic keywords. Near the known binding sites for TATA-binding protein (TBP) in human gene promoters, we found aggressiveness-related candidate SNP markers, including rs1143627 (associated with higher aggressiveness in patients undergoing cytokine immunotherapy), rs544850971 (higher aggressiveness in old women taking lipid-lowering medication), and rs10895068 (childhood aggressiveness-related obesity in adolescence with cardiovascular complications in adulthood).

**Conclusions:**

After validation of these candidate markers by clinical protocols, these SNPs may become useful for physicians (may help to improve treatment of patients) and for the general population (a lifestyle choice preventing aggressiveness-related complications).

**Electronic supplementary material:**

The online version of this article (doi:10.1186/s12864-016-3353-3) contains supplementary material, which is available to authorized users.

## Background

Ethologists define aggressive behavior as a hereditary behavioral pattern performing functions that are important for preservation of the species, namely, defense of the territory, progeny, and shelter and establishment of social-hierarchical relationships within society [1]. Moreover, in many species of animals, low aggressiveness leads to decreased fitness of an individual in particular and problems with reproduction of a population in general [2]. Nonetheless, the opposite extreme—increased aggression among animals of the same species—also has negative consequences, for example, infanticide. In social species of animals, the main mechanism that controls aggressiveness and restrains it within optimal limits is the hierarchical structure of relationships in society. Thus, aggressive behavior has adaptive nature, and its reaction norm is fixed by natural selection [2]. On the other hand, in modern human society, uncontrolled manifestation of aggression is becoming a leading social problem [3–5]. Researchers of aggressive behavior in people classify aggression into several types, which include the impulsive type (caused by external stimuli) and the pathological type [6]. The latter is a symptom of some affective and anxiety disorders. Nonetheless, expression of aggressiveness in real actions of an individual does not depend on its primary causes and manifests itself as physical or verbal aggression [7]. As shown in many experiments on selection for aggressive behavior [8], the latter is an inherited trait, whose phenotypic variability is also influenced by genetic factors [9]. In addition, environmental factors and endogenous ones are so tightly inter-related that research into aggressive human behavior unites the efforts of clinicians, pharmacists, physiologists, geneticists, psychologists, bioinformaticians, pedagogues, sociologists, legal scholars, economists, and other relevant experts, e.g., specialists on insurance, management, health care, law enforcement, and environmental protection. Despite the large number of studies on human aggressiveness, specific genes determining this type of behavior have not been identified to date. The complexity of the problem stems from multifactorial neuroendocrine physiological regulatory mechanisms that are based on genetic systems such as epigenetic regulation of aggressive behavior. For this reason, genome-wide studies of this vitally important form of human behavior are only at the rudimentary stage (e.g., [10]).

One of the biggest modern scientific projects—1000 Genomes [11]—involves identification of SNPs on the whole-genome scale and storing them in the dbSNP database [12], which is an integral part of the reference human genome, which represents the ancestral alleles of all SNPs and, thus, is constantly refined. Taken together with others public parts of the reference human genome such as the Ensembl database [13] and the Web service UCSC Genome Browser [14], dbSNP allows investigators to design, for instance, experiments on gene knockouts in animals designed for research on phenotypic consequences of SNPs as well as for detection of perturbations of gene networks during disorders and under the influence of therapeutic strategies [15].

Biomedical SNP markers represent differences between an individual human genome and the reference human genome; these markers can help to improve a medical treatment [16], to prevent complications of a treatment [17], and to predict comorbidities within the framework of postgenomic predictive preventive personalized medicine [18]. Clinical comparison between cohorts of patients with a given disease and healthy volunteers (as a control) allows researchers to identify SNPs whose allele frequencies significantly separate them from one another as the markers of the above condition (e.g., see [19]). Computer-based analysis of hundreds of millions of unannotated SNPs identified by the 1000 Genomes project [11] may accelerate the clinical search for biomedical SNP markers [20, 21]. Many Web services [22–39] facilitate the bioinformatic search for candidate SNP markers in terms of ranking of unannotated SNPs by their similarity to biomedical SNP markers. These tools take into account whole-genome maps of genes [13, 14], protein-binding sites, interchromosomal contacts, nucleosomes, and transcripts either in health [40], during infection [41] (or other disease [42]), or after treatment [43]. According to the Central Limit Theorem, the accuracy of this similarity-based search for candidate SNP markers increases with the increase in the number of genome-wide maps [44].

Within this mainstream approach, SNPs located in protein-coding gene regions [45] seem to be more informative in the case of monogenic diseases because of the invariant types of disruption in both structure and function of the altered protein [46], whereas SNPs located in regulatory gene regions appear to be more likely to be associated with polygenic treats and disorders, e.g., aggressiveness. With this in mind, the regulatory SNPs in binding sites for TATA-binding protein (TBP) seem to be best studied due to their fixed locations within the narrow region [–70; –20] upstream of the transcription start sites of protein-coding genes in the human genome [47, 48]. Because model animals with a null-mutation [49] or a knockdown of TBP [50] are always inviable, SNPs in TBP-binding sites may be vital and, thus, most promising for computer-based predictions of candidate SNP markers of polygenic treats such as aggressiveness in this study.

Earlier, we developed the public Web service SNP_TATA_Comparator (http://beehive.bionet.nsc.ru/cgi-bin/mgs/tatascan/start.pl) [51, 52] for estimation of the statistical significance (Fisher’s Z-score) of the difference between ancestral and minor SNP variants of a given TBP-binding site in terms of the expression change of the gene whose promoter contains this site [53]. This estimation was explored in detail by our experiments in vitro under both equilibrium [54] and nonequilibrium [55] conditions of the electrophoretic mobility shift assay (EMSA). Furthermore, we verified these estimates using two modern tools of real-time assays, such as a ProteOn™ XPR36 biosensor (Bio-Rad Lab) [56] and an SX.20 spectrometer (Applied Photophysics) [57]. In addition, we tested these estimates using independent data of over 100 experiments by others [58–65]. That is why we apply this approach to studies of unannotated SNPs detected by the 1000 Genomes project [11] which are less known at present. Recently, we predicted candidate SNP markers of complications of hereditary diseases in obesity [66], of autoimmune comorbidities of these diseases [67], and of circadian rhythm disorders [68].

In the present work, we extended the use of our Web service [51, 52] to unannotated SNPs near known SNP markers of monogenic diseases in TBP-binding sites of human gene promoters. Among them, we selected candidate SNP markers of aggressiveness-related complications of these diseases. After validation of these candidate markers by clinical protocols, these SNPs may become useful for physicians (i.e., may help them to improve treatment of patients) and for the general population (e.g., may help to choose a lifestyle preventing aggressiveness-related comorbidities and complications) within the framework of postgenomic predictive preventive personalized medicine [18].

## Results

Tables 1, 2, 3, and 4 show the results obtained using our Web service SNP_TATA_Comparator [51, 52] for the 68 biomedical and candidate SNP markers in the TBP-binding sites of human gene promoters [52] (see Methods: Additional file 1: Supplementary Method). Let us first review in more detail only one human gene in order to briefly describe all the others.

**Table 1:** Candidate SNP markers of aggressiveness as an adverse effect of medical treatments (these markers may change the TBP–promoter affinity)

Gene	dbSNP [12] or see [Reference]	5′ flank	$\frac{\mathbf{wt}}{mut}$	3′ flank	K_D_, nM		Z-score		Known diseases (observations) [Reference] *or hypothetical ones in the case of the candidate SNP markers predicted by us in [this work] (see Methods: Additional file* 2 *: Figure S1)*	[Ref] or *[this work]*
					$\frac{\mathbf{wt}}{mut}$	Δ	Z	α		
*GH1*	rs11568827	aggggccagg	$\frac{\mathbf{g}}{-}$	tataaaaagg	$\frac{\mathbf{1.5}}{1.4}$	=	1	-	Short stature (unknown TF-binding site damaged rather than TATA box)	[69]
									*(hypothetically) low aggression of patients during growth hormone treatment (case review); short stature and aggressiveness co-exist in Smith-Magenis syndrome, Dubowitz syndrome, Floating-Harbor syndrome; and females with constitutional short stature are more aggressive than the ones with Turner syndrome; children and adolescents with hypopituitarism have short stature and show a tendency to avoid aggressiveness (case reviews)*	*[this work]* [70–75]
	rs796237787	gaaggggcca	$\frac{\mathbf{g}}{-}$	ggtataaaaa	$\frac{\mathbf{1.5}}{1.4}$	=	1	-		
	rs768454929	agggtataaa	$\frac{\mathbf{a}}{c}$	agggcccaca	$\frac{\mathbf{1.5}}{2.6}$	↓	7	10^−6^		
	rs761695685	gccagggtat	$\frac{\mathbf{a}}{g}$	aaaagggccc	$\frac{\mathbf{1.5}}{5.8}$	↓	19	10^−6^		
	rs774326004	ccagggtata	$\frac{\mathbf{a}}{t}$	aaagggccca	$\frac{\mathbf{1.5}}{0.9}$	↑	7	10^−6^	*(hypothetically) higher aggression of a mentally unstable patient during growth hormone treatment so that lithium (Li) and other antiaggression medication may be required (a retrospective clinical case review)*	*[this work]* [70]
	rs777003420	aaggggccag	$\frac{\mathbf{g}}{t}$	gtataaaaag	$\frac{\mathbf{1.5}}{1.3}$	↑	3	0.05		
*IL1B*	rs1143627	ttttgaaagc	$\frac{\mathbf{c}}{t}$	ataaaaacag	$\frac{\mathbf{5}}{2}$	↑	15	10^−6^	Greater body fat; gastric cancer, hepatocellular carcinoma, non-small cell lung cancer, gastritis and gastric ulcer, Graves’ disease, major recurrent depression, and also, *(hypothetically) higher aggressiveness in patients who receive cytokine immunotherapy (clinical retrospective review) as well as Graves’ disease and aggressiveness are consequences of regular hemodialysis*	[77–83], *[this work]* [84] [85]
	rs549858786	tgaaagccat	$\frac{\mathbf{a}}{t}$	aaaacagcga	$\frac{\mathbf{5}}{6}$	*↓*	*8*	*10* ^*−6*^	*(hypothetically) less aggressive traits in patients who receive cytokine immunotherapy or regular hemodialysis (clinical retrospective review)*	*[this work],*[84]

Notes: hereinafter, TBP, TATA-binding protein; TATA-box, the canonical TBP-binding site; wt, ancestral allele; mut, minor allele; K_D_, an estimate [52] of the dissociation constant (K_D_) of the TBP-DNA complex in vitro [53]; Δ, the expression change in comparison with the norm: overexpression (↑), underexpression (↓), norm (=); *Z* Z-score; α = 1 – p, significance, where p is probability (Fig. 1); TF, transcription factor; ALS, amyotrophic lateral sclerosis

**Table 2:** Candidate SNP markers of aggressiveness-related drug responses (these markers may significantly change the TBP–promoter affinity)

Gene	dbSNP [12] or see [Reference]	5′ flank	$\frac{\mathbf{wt}}{mut}$	3′ flank	K_D_, nM		Z-score		Known diseases (observations) [Reference] *or hypothetical ones in the case of the candidate SNP markers predicted by us in [this work] (see Methods: Additional file* 2 *: Figure S1)*	[Ref] or *[this work]*
					$\frac{\mathbf{wt}}{mut}$	Δ	Z	α		
*SOD1*	rs7277748	ggtctggcct	$\frac{\mathbf{a}}{g}$	taaagtagtc	$\frac{\mathbf{7}}{2}$	↑	17	10^−6^	Familial amyotrophic lateral sclerosis (ALS), and also, (hypothetically) *aggressiveness is a memantine response in Alzheimer's disease on the basis of this drug’s success in the case of ALS; aggressiveness at late stages of either ALS or traumatic encephalopathy [clinically similar to ALS]; lesser male–male aggression (SOD1-deficient mouse males)*	[86], *[this work]* [87–90]
*StAR*	rs16887226	cagccttcag	$\frac{\mathbf{c}}{t}$	gggggacatt	$\frac{\mathbf{10}}{10}$	=	1	-	Diabetic hypertension (unknown TF-binding site damaged, not TATA box)	[91]
									*(hypothetically) lithium (Li) is a common drug against aggressiveness, hypertension, and diabetes (case reviews); old women on lipid-lowering medication become more aggressive, hypertensive, and diabetic; both diabetes and hypertension coexist with aggressiveness in a magnesium (Mg) deficiency, in intermittent explosive disorder, in Alzheimer's disease, in postmenopausal women with multiple medical problems in contrast to reduced aggressiveness in old men regardless of disease and lesser male aggression (a fish model on human behavior);as well as coexistence of aggressiveness, hypertension, and diabetes can elevate risk of nonfatal myocardial infarction; diet has long-term impact on aggressiveness, hypertension, and diabetes; aggressiveness, hypertension, and diabetes are risk factors of cerebrovascular disease, cerebral sclerosis;*	*[this work]* [92–107]
	rs544850971	tcagcggggg	$\frac{\mathbf{a}}{g}$	catttaagac	$\frac{\mathbf{10}}{12}$	↓	5	10^−6^		
*NOS2*	see [110]	gtataaatac	$\frac{\mathbf{t}}{c}$	tcttggctgc	$\frac{\mathbf{2}}{1}$	↑	3	10^−2^	Resistance to malaria, epilepsy risk, and also, *(hypothetically) drug-resistant or childhood epilepsy is associated with aggressiveness; stigma as a critical factor for interictal aggression in epilepsy (clinical review); aggression, hyperactivity, and impaired memory coexist during recurrent spontaneous seizures in epilepsy (rat model), gender-biased complication of excessive lead (Pb) intake manifested as lesser exploration in females and higher aggressiveness in males (mice)*	[108–110] *[this work]* [111] [112–115]

**Table 3:** Candidate SNP markers of aggressiveness as a symptom of hereditary diseases (these markers may change the TBP–DNA affinity)

Gene	dbSNP [12] or see [Reference]	5′ flank	$\frac{\mathbf{wt}}{mut}$	3′ flank	K_D_, nM		Z-score		Known diseases (observations) [Reference] *or hypothetical ones in the case of the candidate SNP markers predicted by us in [this work] (see Methods: Additional file* 2 *: Figure S1)*	[Ref] or *[this work]*
					$\frac{\mathbf{wt}}{mut}$	Δ	Z	α		
*ESR2*	rs35036378	cctctcggtc	$\frac{\mathbf{t}}{g}$	ttaaaaggaa	$\frac{\mathbf{6}}{8}$	↓	5	10^−3^	ESR2-deficient pT1 tumor,	[116]
									*(hypothetically) ESR2-deficient maladaptive aggressive social behaviors caused by bisphenol A and phthalates in children (primary tumors and aggression are well-known consequences of environmental pollution with bisphenol A)*	*[this work]* [117, 118]
	rs766797386	ttaaaaggaa	$\frac{\mathbf{g}}{t}$	aaggggctta	$\frac{\mathbf{6}}{7}$	↓	3	10^−2^		
*HBB*	rs397509430	gggctgggca	$\frac{\mathbf{t}}{-}$	atacaacagt	$\frac{\mathbf{5}}{29}$	↓	34	10^−6^	Malaria resistance, thalassemia, *and also (hypothetically) thalassemia-related higher male–male aggression, socialized aggression, inattention, low IQ, acute psychosis with aggression, impulsiveness as a form of aggressiveness; aggression as a comorbidity in 4-yo and 5-yo girls and in boys with thalassemia in a hospital; aggressiveness as a consequence of regular hemodialysis in a severe form of thalassemia*	[119] *[this work]* [85, 120–125]
	rs33980857	gggctgggca	$\frac{\mathbf{t}}{a,g,c}$	atacaacagt	$\frac{\mathbf{5}}{21}$	↓	27	10^−6^		
	rs34598529	ggctgggcat	$\frac{\mathbf{a}}{g}$	aaagtcaggg	$\frac{\mathbf{5}}{18}$	↓	24	10^−6^		
	rs33931746	gctgggcata	$\frac{\mathbf{a}}{g,c}$	aagtcagggc	$\frac{\mathbf{5}}{11}$	↓	14	10^−6^		
	rs33981098	agggctgggc	$\frac{\mathbf{a}}{g,c}$	taaaagtcag	$\frac{\mathbf{5}}{9}$	↓	10	10^−6^		
	rs34500389	cagggctggg	$\frac{\mathbf{c}}{a,t,g}$	ataaaagtca	$\frac{\mathbf{5}}{6}$	↓	3	10^−2^		
*(HBD)*	rs35518301	caggaccagc	$\frac{\mathbf{a}}{g}$	taaaaggcag	$\frac{\mathbf{4}}{8}$	↓	11	10^−6^		
*HBB*	*rs63750953*	*ctgggcataa*	$\frac{\mathbf{aa}}{-}$	*gtcagggcag*	$\frac{\mathbf{5}}{8}$	*↓*	*9*	*10* ^*−6*^	*(hypothetically)malaria resistance, thalassemia, thalassemia-related higher male–male aggression, socialized aggression, inattention, low IQ, acute psychosis with aggression, impulsiveness as a form of aggressiveness; aggression as a comorbidity in 4-yo and 5-yo girls and in boys with thalassemia in a hospital; aggressiveness as a consequence of regular hemodialysis in a severe form of thalassemia*	*[this work]* [85, 119, 125]
	*rs281864525*	*tgggcataaa*	$\frac{\mathbf{a}}{c}$	*gtcagggcag*	$\frac{\mathbf{5}}{7}$	*↓*	*7*	*10* ^*−6*^		
*(HBD)*	rs34166473	aggaccagca	$\frac{\mathbf{t}}{c}$	aaaaggcagg	$\frac{\mathbf{4}}{8}$	*↓*	*18*	*10* ^*−6*^

**Table 4:** Candidate SNP markers of obesity-related aggressiveness (these markers may change the TBP–promoter affinity)

Gene	dbSNP [12] or see [Reference]	5′ flank	$\frac{\mathbf{wt}}{mut}$	3′ flank	K_D_, nM		Z-score		Known diseases (observations) [Reference] *or hypothetical ones in the case of the candidate SNP markers predicted by us in [this work] (see Methods: Additional file* 2 *: Figure S1)*	[Ref] or *[this work]*
					$\frac{\mathbf{wt}}{mut}$	Δ	Z	α		
*PGR*	rs10895068	gggagataaa	$\frac{\mathbf{g}}{a}$	gagccgcgtg	$\frac{\mathbf{10}}{6}$	↑	8	10^−6^	Endometrial cancer in obese women [*de novo* pathogenic TATA box], *and also (hypothetically) obese school-aged girls show verbal aggressiveness (e.g., victimization); obese females are more aggressive (primate model); obesity and aggression coexist in polycystic ovary syndrome in women with biliary calculi (retrospective clinical reviews); high aggression/rejection in reproductive behavior (mice); low attraction and high aggression against males in females (rabbit model)*	[126], *[this work]* [127–132]
*LEP*	rs201381696	tcgggccgct	$\frac{\mathbf{a}}{g}$	taagaggggc	$\frac{\mathbf{4}}{12}$	↓	17	10^−6^	*(hypothetically) obesity and also, in a 10 yo girl, aggressiveness is a predictive factor for prevention of obesity in adolescence with cardiovascular complications in adulthood, as is the case for 5 yo boys (retrospective review); aberrant maternal behavior, low aggression against an unknown social stimulus and locomotor activity during a high-fat diet (mice); low chance to be dominant due to aggressiveness against subordinates in female social behavior (macaques model), high risks of suicidality, violence, and impulsive aggressiveness in 19–45 yo patients with schizophrenia; higher social aggressiveness in males (rat model); longer survival in aggressive leptin-deficient women with anorexia nervosa*	*[this work],* [115, 134–143]
	rs200487063	tgatcgggcc	$\frac{\mathbf{g}}{a}$	ctataagagg	$\frac{\mathbf{4}}{2}$	↑	6	10^−6^	*(hypothetically) obesity-caused hypertension, and also, lower risk of aberrant maternal behavior, higher aggression against an unknown social stimulus, and locomotion activity on a high-fat diet (mice); higher chance to be dominant due to aggressiveness against subordinates in female social behavior (macaque model); lower risks of suicidality, violence, and impulsive aggressiveness in 19–45 yo schizophrenic patients; lower social aggressiveness in males (rat model)*	*[this work]* [115, 134–142]
	rs34104384	ccgctataag	$\frac{\mathbf{a}}{t}$	ggggcgggca	$\frac{\mathbf{4}}{3}$	↑	4	10^−2^		

### Candidate SNP markers of aggressiveness as an adverse effect of medical treatments

**Fig. 1:**
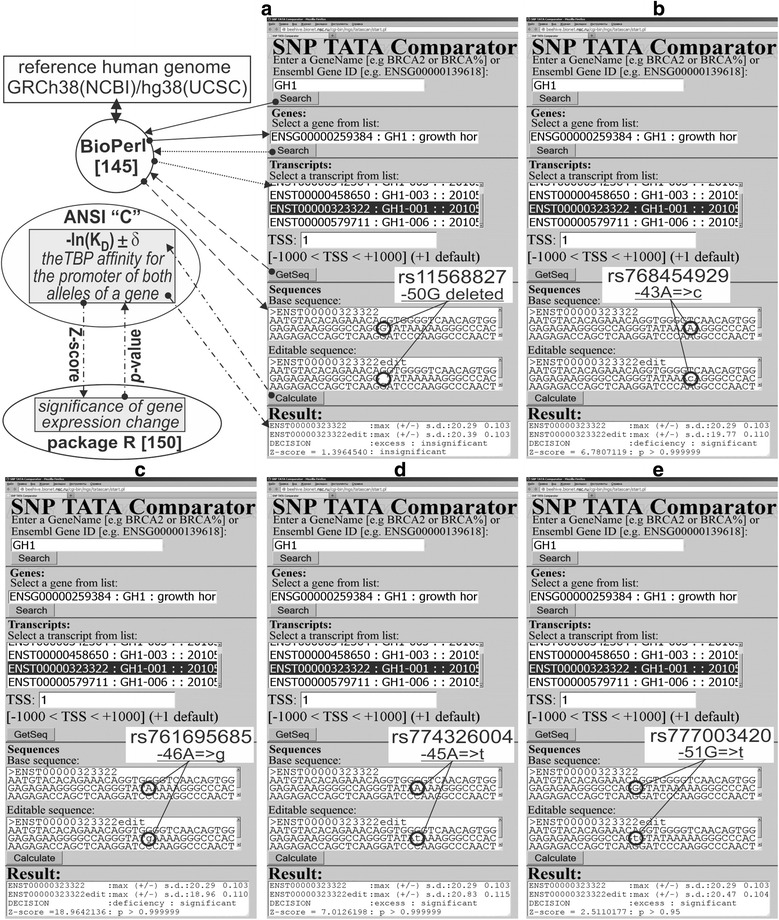
The result produced by SNP_TATA_Comparator [51, 52] for aggressiveness-related SNP markers of the human *GH1* gene. **a** rs11568827, **b** rs768454929, **c** rs761695685, **d** rs774326004, and **e** rs777003420. Solid, dotted, and dashed arrows denote BioPerl queries [145] to the reference human genome. *Dash*-and-*dot* arrows indicate statistical significance estimates for the alteration of gene expression by the minor allele (in comparison with the ancestral allele) using the R package [150]. *Circles* indicate the alleles marked by their dbSNP ID [12]

The human *GH1* gene (growth hormone 1, synonym: somatotropin) contains a biomedical SNP marker (rs11568827) of short stature [69]. According to the results of electrophoretic mobility shift assay (EMSA) [69], this SNP reduces this gene’s expression because it damages the binding site for an unknown transcription factor rather than the TBP-binding site (Table 1. The prediction of our Web service [51, 52] was consistent with these independent experimental data (Fig. 1a: text box “Results”, line “Decision” contains the label “insignificant”).

First, using the primary keyword search (hereinafter: see Methods, Additional file 2: Figure S1: two boxes outlined with a dashed line), we found the retrospective clinical review [70] showing that a *GH1* deficiency is a biochemical marker of lesser aggression of mentally unstable patients during growth hormone treatment when the dose of the additional lithium (Li)-based or others antiaggression medication may be reduced). Next, using the secondary keyword search (hereinafter: see Methods: Additional file 2: Figure S1: one box outlined with a dotted line), we found the retrospective and clinical case reviews indicating that short stature and aggressiveness coexist in Smith-Magenis syndrome [71, 72], Dubowitz syndrome [73], and Floating-Harbor syndrome [74]. In addition, women of constitutionally short stature are more aggressive than the ones with Turner syndrome [75]. In contrast, children and adolescents with hypopituitarism have short stature and show a tendency to avoid aggression.

On the basis of all the above reasons together with our recent hypothesis on “how SNP may change the apparent biological activity of drugs inhibiting target genes” [76], we propose rs11568827 as a candidate SNP marker associated with a lesser dose of an additional antiaggression drug during growth hormone treatment of mentally instable patients (Table 1).

Two base pairs away from a known biomedical marker (rs11568827), we found an unannotated SNP (rs796237787), which also represents a deletion of G. For this SNP, our Web service predicted the same change in the same TBP-binding site (Table 1). Therefore, we also propose rs796237787 as a candidate SNP marker of the same pathologies.

In addition, we found two unannotated SNPs (rs768454929, and rs761695685) that significantly damage the TBP-binding site in question, and thus reduce expression of the *GH1* gene, as is the case for the known biomedical marker rs11568827. On this basis, we propose rs768454929 and rs761695685 as candidate SNP markers of the same disorders.

Finally, immediately upstream of the known biomedical marker rs11568827, we identified two unannotated SNPs (rs777003420 and rs774326004) for which our Web service predicted a significant increase in the affinity of TBP for the promoter of the *GH1* gene, and accordingly, increased expression of this gene. That is why we propose rs777003420 and rs774326004 as candidate SNP markers associated with a higher dose of an additional antiaggression drug during growth hormone treatment of mentally instable patients.

The human *IL1B* gene (interleukin 1β) has an SNP marker (rs1143627) of a wide variety of human diseases such as Graves’ disease [77], major recurrent depression [78], greater body fat in older men [79], non–small cell lung cancer [80], hepatocellular carcinoma [81], gastric cancer [82], gastric ulcer, and chronic gastritis [83] (Table 1). Previously, we experimentally verified in depth the predictions of our Web service [51, 52] for this SNP (rs1143627) by EMSA under both equilibrium [54] and nonequilibrium [55] conditions. First, our primary keyword search pinpointed a retrospective clinical review [84] about higher aggressiveness in patients who receive cytokine immunotherapy. Next, the secondary keyword search, produced a clinical case of regular hemodialysis that resulted in aggressiveness and Graves’ disease at the same time [85]. Therefore, a human disease associated with the known SNP marker rs1143627 co-occurs with aggressiveness. For this reason, we predicted that this well-known biomedical SNP marker (rs1143627) can also be considered a candidate SNP marker of higher aggressiveness in patients receiving either cytokine immunotherapy or hemodialysis.

Near this biomedical SNP marker rs1143627, we found the unannotated SNP rs549858786, which can significantly reduce the human *IL1B* gene expression. That is why, rs1143627 may be a candidate SNP marker of lesser aggressiveness in patients undergoing either cytokine immunotherapy or hemodialysis.

### Candidate SNP markers of aggressiveness-related drug responses

The human *SOD1* gene (superoxide dismutase 1, synonym: Cu/Zn superoxide dismutase): its promoter contains a known SNP marker (rs7277748) of familial amyotrophic lateral sclerosis (ALS) [86]: this SNP causes overexpression of this gene. Our primary keyword search yielded laboratory findings on higher intermale aggression in a murine model completely deficient in the *SOD1* gene [87] (Table 2).

As for coexistence of aggressiveness and ALS, our secondary keyword search identified three articles on aggressiveness at late stages of ALS [88], in ALS with frontotemporal dysfunction [89], and in chronic traumatic encephalopathy whose signs and symptoms are clinically similar to those of ALS [89]. In addition, aggressiveness is a complication of the memantine-based treatment of Alzheimer’s disease which was used due to success of the memantine-based treatment of ALS [90]. For all these reasons, we predicted that the known SNP marker rs7277748 can additionally be a candidate SNP marker of lesser male–male aggression, significant aggressiveness in ALS and in patients with Alzheimer’s disease during memantine-based treatment.

The human *StAR* gene (steroidogenic acute regulatory protein, synonym: cholesterol trafficker) has a known SNP marker (rs544850971) of hypertension in diabetes [91] (Table 2). This SNP destroys a binding site for an unknown transcription factor (not a TBP-binding site) and thereby causes underexpression of the human StARs gene. Here, using a primary keyword search, we found a laboratory finding of lesser male aggression in a StAR-deficient fish model of human behavior [92]. In the case of our secondary keyword search, we found a number of articles [93–107] describing co-occurrence of aggressiveness, hypertension, and diabetes. As an example, old women on lipid-lowering medication become more aggressive and hypertensive and develop signs of diabetes [100]. Therefore, we propose the known SNP marker rs16887226 (hypertensive diabetes) as a candidate SNP marker of aggressiveness in many clinical and nonclinical cases listed in Table 2.

Near the well-known biomedical SNP marker rs16887226, we found the unannotated SNP rs544850971. Next, we predicted using our Web service [51, 52] that this SNP can also cause underexpression of the human StAR gene, and, then, we proposed rs544850971 as a candidate SNP marker of the same diseases.

The human *NOS2* gene (inducible nitric oxide synthase 2) contains an SNP marker of resistance to malaria [108] and epilepsy [109] where the –51 T → C substitution (relative to the transcription start site of this gene [110]) causes NOS2 overexpression [108–110]. Our primary keyword search yielded laboratory data on a gender-biased complication of excessive lead (Pb) intake (a murine model): lesser exploration in females and higher aggressiveness in males [111]. As for the secondary keyword search, it produced over 1,500 original articles on the co-occurrence of aggressiveness and epilepsy; here, we cite only the most interesting studies in our opinion. For example, both drug-resistant epilepsy and childhood epilepsy are associated with aggressiveness [112, 113] as well as a perceived stigma is a critical factor of interictal aggression, hyperactivity, and impaired memory during recurrent spontaneous seizures in epilepsy [114, 115]. On this basis, we predicted that –51 T → C substitution within the known TATA-box of the human *NOS2* gene can be a candidate SNP marker of higher aggressiveness in males under the influence of environmental pollution with Pb as one can see in (Table 2).

### Candidate SNP markers of aggressiveness as a symptom of hereditary diseases

The human *ESR2* gene (estrogen receptor β) promoter contains a known SNP marker (rs35036378) of a primary ESR2-deficient pT1 tumor whose development can lead to breast cancer without proper preventive treatment [116] (Table 3).

Using a primary keyword search, we uncovered a clinical case of maladaptive social behaviors (e.g., aggression) caused by bisphenol A and phthalates in children [117]. Moreover, our secondary keyword search supported these striking findings by a retrospective clinical review showing that both primary tumors and aggression among the many behavioral disorders are well-known consequences of environmental pollution with bisphenol A [118]. With this in mind, we suggest the SNP marker rs35036378 (a primary ESR2-deficient pT1 tumor) as a candidate SNP marker of childhood aggressiveness caused by bisphenol A.

Near this biomedical SNP marker, we found an unannotated SNP (rs766797386) that can also reduce the human ESR2 gene expression as it was predicted by our Web service [51, 52]. Thus, we recommend to verify them as candidate SNP markers (rs766797386) of the above-mentioned human disorders.

The human *HBB* and *HBD* genes (β- and δ-chains of hemoglobin, respectively) have the largest number of known SNP markers (rs34500389, rs33981098, rs33980857, rs34598529, rs33931746, rs397509430, and rs35518301) of resistance to malaria and thalassemia (Cooley’s anemia) [119] (Table 3). According to output of a primary keyword search [120–122], a hemoglobin deficiency is associated with higher intermale aggression, socialized aggression, inattention, low IQ, acute psychosis with aggression, and also with aggression in 4- and 5-year-old girls. Similarly, our secondary keyword search showed that thalassemia increases the risk of aggressiveness (impulsiveness) [123, 124] and that aggressiveness is a comorbidity in hospitalized boys with thalassemia [125]. For these reasons, we nominate these biomedical SNP markers as candidate SNP markers of aggressiveness in Cooley’s anemia.

Near these known SNP markers of hereditary diseases, we found three unannotated SNPs (rs63750953, rs281864525, and rs34166473) which can cause a hemoglobin deficiency in humans according to our Web service predictions. Thus, we propose them as candidate SNP markers of aggressiveness as a complication of thalassemia.

### Candidate SNP markers of obesity-related aggressiveness

The human *PGR* gene (progesterone receptor) has the biomedical SNP marker rs10895068 of endometrial cancer in obese women [126] caused by this gene’s overexpression (Table 4).

Our primary keyword search retrieved the laboratory findings about female reproductive behavior where the progesterone receptor excess increases aggression toward/rejection of males in a murine model [127] and aggression against males in a rabbit model [128]. As for our secondary keyword search, it produced a large number of articles showing co-occurrence of aggressiveness and obesity in women [129–132]. For instance, obese school-aged girls are predisposed to verbal aggressiveness (e.g., victimization) [129]; in addition, obesity and aggression coexist in polycystic ovary syndrome [131] and in women with biliary calculi [132]. Accordingly, we predict rs10895068 to be a candidate SNP marker of gender-biased aggressiveness in obese women.

The human *LEP* gene (leptin; synonyms: obesity factor with acronym *OB*) contains a candidate SNP marker (rs201381696) of obesity (reducing this gene’s expression) as well as candidate SNP markers (rs200487063 and rs34104384) of obesity-induced hypertension caused by overexpression of this gene as we have predicted *in silico* and verified in vitro in our previous work [66] (Table 4).

**Fig. 2:**
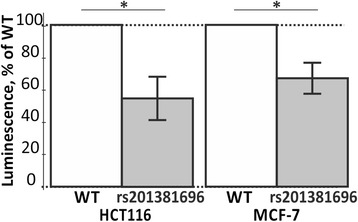
Cell culture verification of the candidate SNP marker rs201381696 in human cell lines transfected with the pGL 4.10 vector carrying a reporter *LUC* gene. *Open* bars, ancestral allele (wild type, WT); *gray* bars, minor allele; HCT116, a human colon adenocarcinoma cell line as an example of basal expression of the human *LEP* gene; MCF-7, a cell line of the human mammary gland epithelium carcinoma as an example of tissue-specific expression of this gene. The height of the gray bars and their error bars correspond to the mean estimates and their standard deviations calculated from at least three independent measurements. Asterisks indicate a statistically significant difference at the confidence level α < 0.05

In this work, we experimentally confirmed (in cell culture) the rs200487063-caused deficient expression of the *LEP* gene using the pGL 4.10 vector (the reporter gene LUC for luciferase; see Methods: Cell culture, transfection, and reporter assays) whose expression can be seen in Fig. 2.

This figure shows that the rs200487063-caused significant downregulation of reporter gene *LUC* is approximately twofold in both cell lines: HCT116 (human colon adenocarcinoma exemplifying basal expression of the human *LEP* gene) and MCF-7 (carcinoma of the human mammary gland epithelium exemplifying tissue-specific expression of this gene), at α < 0.05 according to Student’s *t*-test (asterisks in Fig. 2). As one can see in Fig. 2, there are no differences in the effects of this candidate SNP marker (rs200487063) between the basal and tissue-specific mode of the human *LEP* gene expression in our study, in agreement with a well-known independent experiment on multiple promoter models [133].

That is why we then conducted our primary keyword search for publications associating aggressiveness with a significant deficiency in this gene’s expression; this search produced a large number of research papers and review articles on this topic [115, 134–143]. As an example, our secondary keyword search identified a retrospective review [135] showing that high aggressiveness in 10-year-old girls is a statistically significant predictive factor of obesity in adolescence (*p* < 0.0005) and cardiovascular complications in adulthood; the same is true for 5-year-old boys. Furthermore, a leptin deficiency in a murine model causes aberrant maternal behavior, lower aggression against an unknown social stimulus, and increased locomotor activity during a high-fat diet [136]. In addition, a clinical case review [143] revealed longer survival in aggressive leptin-deficient women with anorexia nervosa. On the basis of these data, we expanded our prediction [66] on the obesity-related candidate SNP markers rs201381696, rs200487063, and rs34104384 to our prognosis that these SNPs can also be candidate SNP markers of aggressiveness in obesity.

## Discussion

Because the TBP-binding site is one of the best-studied regulatory sequences within the human genome [48], we limited our research to SNPs altering the human gene expression via statistically significant changes in the TATA-binding protein’s affinity for human gene promoters. Using our Web service SNP_TATA_Comparator [51, 52], we analyzed 493 SNPs located within [−70; −20] proximal promoter regions of 33 human genes and found only 28 aggressiveness-related candidate SNP markers (6%). Each of them can alter expression of one of 10 human genes via significant changes in the TBP-binding affinity of promoters of these genes, as we deduced from our results shown in Tables 1, 2, 3 and 4. This finding does not mean that the other 465 of the 493 SNPs (94%, data not shown) cannot be considered aggressiveness-related candidate SNP markers; they may at least alter transcription factor-binding sites (e.g., rs11568827, rs796237787, and rs16887226). To conduct this kind of analysis for any of them, one can find a number of public Web services [22–39] for in-depth studies on other molecular mechanisms behind the effects of SNPs on human health; these services’ research capabilities can be enhanced when they are used together with our Web service SNP_TATA_Comparator [51, 52].

It should be emphasized that known SNP markers of monogenic diseases *cause* these diseases, whereas candidate SNP markers of polygenic diseases whose symptoms include aggressiveness can only serve as genomewide informative landmarks suggestive of either increased or decreased risk of aggressiveness (in these diseases relative to the norm) among patients with the minor alleles of these SNPs [67]. For example, here we predicted a candidate SNP marker (rs201381696) of aggressiveness in obesity. Using this whole-genome landmark, parents of an aggressive 10-year-old girl with a minor allele of rs201381696 may choose a diet and a physical exercise regimen for their daughter to prevent her obesity in adolescence and cardiovascular complications in adulthood; the same approach is applicable to 5-year-old boys. Similarly, using our suggested candidate SNP marker (rs1143627) of higher aggressiveness in patients receiving cytokine immunotherapy, a physician can prescribe an antiaggression medication together with cytokine immunotherapy to a patient carrying a minor allele of this SNP. In addition, according to our prediction of the candidate SNP marker (rs35036378) of childhood aggressiveness caused by bisphenol A, parents may look into the presence of this compound in plastic toys of their child if he/she has the minor allele of this SNP. Furthermore, using the candidate SNP marker of higher aggressiveness in males subjected to environmental pollution with Pb [the –51 T → C substitution in the human *NOS2* gene promoter], people with a minor allele of this SNP can modify their lifestyle to minimize their contact with materials containing lead.

In this study, we encountered a huge number of clinical cases, retrospective reviews, research articles, laboratory data, and empirical findings—on aggressiveness in various life situations—from clinicians, pharmacists, physiologists, geneticists, psychologists, bioinformaticians, pedagogues, sociologists, legal scholars, economists, and other relevant experts such as specialists on insurance, management, health care, law enforcement, and environmental protection. The gigantic scale, multidisciplinary nature, complexity, and disarray of this information pool may hinder the use of this vital knowledge for broad practical applications in the general population. As shown in Tables 1, 2, 3 and 4, candidate SNP markers of aggressiveness seem to be promising whole-genome landmarks around which researchers can organize existing knowledge about this integral characteristic of the genome as a whole; this characteristic reflects the individual mobilization potential of the human body. The usable portions of this knowledge may be directly applicable to people carrying a minor allele of such SNPs.

Finally, each aggressiveness-related candidate SNP marker predicted in this work needs comprehensive verification under various in vitro and in vivo experimental conditions, as well as in clinical protocols involving representative cohorts of patients with the corresponding diseases and healthy volunteers (as a control). After that, such SNP markers will become practically applicable. To facilitate the validation, for each predicted candidate SNP marker we show a quantitative parameter: the equilibrium dissociation constant (K_d_) for binding of human TATA-binding protein to 26-bp synthetic duplex DNA identical to the SNP in question (as the prediction of our Web service, expressed in “nanomoles per liter” units, nM; Tables 1, 2, 3 and 4). These additional data are intended for optimization of experimental and clinical conditions during verification of our predictions before their practical use.

## Conclusions

If these aggressiveness-related candidate SNP markers are validated by clinical protocols, these whole-genome landmarks may become useful for physicians (may help to optimize treatment of patients) as well as for the general population (may help to choose a lifestyle preventing aggressiveness-related comorbidities and complications).

## Methods

### Cell culture, transfection, and reporter assays

Cell lines HCT116 (human colon adenocarcinoma) and MCF-7 (carcinoma of the human mammary gland epithelium) were cultivated in a complete medium consisting of Dulbecco’s modified Eagle’s medium/Nutrient mixture F-12 Ham, supplemented with 10% (v/v) of fetal bovine serum (Sigma) and penicillin (100 U/mL) and streptomycin (100 mg/mL) (BioloT). The cultures were maintained at 37 °C in a humidified atmosphere containing 5% of CO_2_ until the desired level of confluence. All the experiments were performed at 80–85% confluence. Oligonucleotides corresponding to ancestral and minor alleles of the predicted candidate SNP marker rs201381696 (Table 4) were cloned into the pGL 4.10 vector (Promega, USA) and cotransfected with pRL-TK using Screen Fect A (InCella) as described by Wolfe and the colleagues [144]. After that, the cells were cultured in 6-well plates for 24 h. Luciferase activity was measured by means of the Dual-Luciferase Reporter Assay kit (Promega).

### DNA sequence analysis *in silico*

We analyzed DNA sequences of the human gene promoters retrieved from the human reference genome using the standard BioPerl library [145] via our Web service [51, 52], (Fig. 1) in the case of ancestral alleles of SNPs that were analyzed and those manually corrected on the basis of the description of these alleles from the database dbSNP [12]. For each DNA sequence, we calculated the maximal value and its standard deviation –ln(K^0^_D_) ± δ of the affinity of TBP for the [–70; −20] promoter region (where all the known sites are located) using our Web service [51, 52] as described in Additional file 1 [146–150].

### Keyword search within the NCBI databases

For each case of predicted significant overexpression or underexpression of the human genes (as clinically relevant biochemical markers), we conducted a manual two-step keyword search in the NCBI databases [151] as described in detail elsewhere [152] and as depicted schematically in Additional file 2: Figure S1. In this figure, two boxes consisting of a dashed line depict the primary keyword search for diseases whose symptoms include aggressiveness and whose known biochemical markers correspond to the gene expression alteration caused by the SNP being considered.

In addition, in Additional file 2: Figure S1, there is a box outlined with a dotted line that depicts the secondary keyword search for co-occurrence of the aggressiveness-related disease found by the primary keyword search and the hereditary disease clinically associated with this SNP. Our heuristic interpretation of each aggressiveness-related candidate SNP marker in accordance with a significant alteration of expression of a human gene is listed in the second rightmost column of Tables 1, 2, 3 and 4; the supporting information consisting of clinical cases, retrospective reviews, empirical data, laboratory observations, and published hypotheses are cited in the rightmost column.

## Additional files

Additional file 1: Supplementary method. A quantitative sequence-based prediction of binding affinity of TATA-binding protein (TBP) for a human gene promoter. (PDF 161 kb)
Additional file 2: Figure S1. A flow chart of the keyword search for aggressiveness-related diseases whose biochemical markers correspond to an alteration in expression of the human gene under study containing the candidate SNP marker of interest. Legend: two boxes consisting of a dashed line depict the primary keyword search for diseases whose symptoms include aggressiveness; the box outlined with a dotted line depicts the secondary keyword search for co-occurrence of the aggressiveness-related disease found by the primary keyword search and the hereditary disease clinically associated with the SNP in question. (PNG 385 kb)

## Abbreviations

ALS: Amyotrophic lateral sclerosis
EMSA: Electrophoretic mobility shift assay
K_d_: Equilibrium dissociation constant
ln: Natural logarithm
mut: Minor allele of SNPs
SNP: Single nucleotide polymorphism
TBP: TATA-binding protein
TF: Transcription factor
TSS: Transcription start site
WT: Wild type (norm)
