# Supplementary material for: Candidate SNP markers of aggressiveness-related complications and comorbidities of genetic diseases are predicted by a significant change in the affinity of TATA-binding protein for human gene promoters
Source: BMC Genomics. 2016 Dec 28;17(Suppl 14):995. doi: 10.1186/s12864-016-3353-3 (PMC5249025; doi:10.1186/s12864-016-3353-3)
Supplement: Additional file 1: — Supplementary method. A quantitative sequence-based prediction of binding affinity of TATA-binding protein (TBP) for a human gene promoter. (PDF 161 kb) [file 12864_2016_3353_MOESM1_ESM.pdf]

Additional file 1: Supplementary Method**.**

A quantitative sequence-based prediction of the TATA-binding protein (TBP) binding affinity for the human gene promoter

The initializing data are the 90-bp DNA sequence $\{s_{-90}\ldots s_i\ldots s_{-1}\}$ immediately upstream of the transcription start site (TSS, s_0) (where $s_i \in \{a, c, g, t\}$).

We used the linear approximation of the three-step molecular mechanism of TBP's binding to the [–70; –20] region of the eukaryotic gene promoters—e.g.: (i) TBP slides along DNA ↔ (ii) TBP stops at a potential TBP-binding site ↔ the DNA helix bends to the 90° angle and stabilizes the local TBP-promoter complex—as follows:

$$-\ln(K_D) = 10.9 - 0.2\ \{\ln(K_{SLIDE}) + \ln(K_{STOP}) + \ln(K_{BEND})\}, \qquad \textbf{(1)}$$

where 10.9 (ln units) is nonspecific TBP-DNA affinity (10^{-5} M), 0.2 is the stoichiometric coefficient, and K_{STOP} is our heuristic estimate of the equilibrium constant of the second step of the TBP stops at a TBP-binding site (the maximal score value of Bucher's position-weight matrix, the commonly accepted criterion of the canonical form of a TBP-binding site [146]); K_{SLIDE} is our heuristic estimate of the equilibrium constant of the first step of the TBP sliding along DNA; we estimated its value empirically as

$$-\ln(K_{SLIDE}) = MEAN_{15bp}\ \{0.8[TA]_{3'HALF} - 3.4MGW_{CENTER} - 35.1\},$$

where $[TA]_{3'HALF}$ is the frequency of dinucleotide TA within the 3′ half of the sequence being analyzed; MGW_{CENTER} is the arithmetical mean width of the mutor groove of the DNA helix [147]; 0.8, –3.4, and –35.1 are linear regression coefficients taken from our original experimental data [148].

In Eq. **(1)**, K_{BEND} is our heuristic estimate of the equilibrium constant at the third step of DNA helix bending; we estimated its value empirically as

$$-\ln(K_{BEND}) = MEAN_{TATA\text{-}box}\ \{0.9[TA, AA, TG, AG]_{FLANK} + 2.5[TA, TC, TG]_{CENTER} + 14.4\},$$

where 0.9, 2.5, and 14.4 are linear regression coefficients calculated from our original experimental data [149]; $MEAN_{TATA\text{-}box}$ is the arithmetic mean value for both DNA strands of the TBP-binding site at the position of the maximal score value of Bucher's position-weight matrix [146].

Using all the 78 possible nucleotide substitutions, $s_{i+j}\rightarrow\xi$, at each j-th position ($-13 \le j \le 12$; 3 × 26) within the 26-bp DNA window centered by i-th position of the promoter DNA under study, we estimated heuristically the standard deviation of the $-\ln[K_D]$ estimates (Eq. 1), namely:

$$\delta = [(\Sigma_{1\le i\le 26}\Sigma_{\xi\in\{a,c,g,t\}}[\ln(K_D(\{s_{i-13}\ldots s_{i+j-1}\xi s_{i+j+1}\ldots s_{i+12}\})/K_D(\{s_{i-13}\ldots s_{i+j-1}s_{i+j}s_{i+j+1}\ldots s_{i+12}\})^2])/78]^{1/2}. \qquad \textbf{(2)}$$

Thus, the prelimutary result of the DNA sequence analysis is the maximal value of $-\ln(K_D) \pm \delta$ among all the possible estimates of TBP's binding affinity for the DNA fragment of 26-bp in length, $\{s_{i-13}\ldots s_i\ldots s_{i+12}\}$ at the i-th position in-between –70 and –20 for both DNA chains (where K_D is the equilibrium dissociation constant expressed in moles per liter; M).

Applying Eqs. **(1–2)** to the cases of two mutor and ancestral alleles of a given gene, $(-\ln(K_D^{(mut)}) \pm \delta_{(mut)})$ and $(-\ln(K_D^{(wt)}) \pm \delta_{(wt)})$, we calculated Fisher's Z-score such as

$$Z = abs[\ln(K_D^{(mut)}/K_D^{(wt)})]/[\delta^2_{(mut)}+\delta^2_{(wt)}]^{1/2}.$$

The statistical package R [150] transformed this Z-score value into the p value of the probability rate of acceptance of the hypothesis "H_0: $-\ln(K_D^{(mut)}) \neq -\ln(K_D^{(wt)})$" (where $\alpha = 1 - p$ is the statistical significance level). At this statistically significant level $\alpha < 0.05$ (i.e., at $p > 0.95$), we made the final decision:

IF {**INEQUALITY** "$-\ln(K_D^{(mut)}) > -\ln(K_D^{(wt)})$" is statistically significant},

THEN {**DECISION** is "there is excessive expression of the mutor allele of a given gene versus the ancestral allele"};

ELSE [**IF** {**INEQUALITY** "$-\ln(K_D^{(mut)}) < -\ln(K_D^{(wt)})$" is statistically significant},

THEN {**DECISION** is "there is lower expression of the mutor allele of this gene versus the ancestral allele"},]

OTHERWISE {**DECISION** is "alteration of the expression of this gene is insignificant"}.
