# Supplementary figures and images for: Candidate SNP markers of aggressiveness-related complications and comorbidities of genetic diseases are predicted by a significant change in the affinity of TATA-binding protein for human gene promoters
Source: BMC Genomics. 2016 Dec 28;17(Suppl 14):995. doi: 10.1186/s12864-016-3353-3 (PMC5249025; doi:10.1186/s12864-016-3353-3)

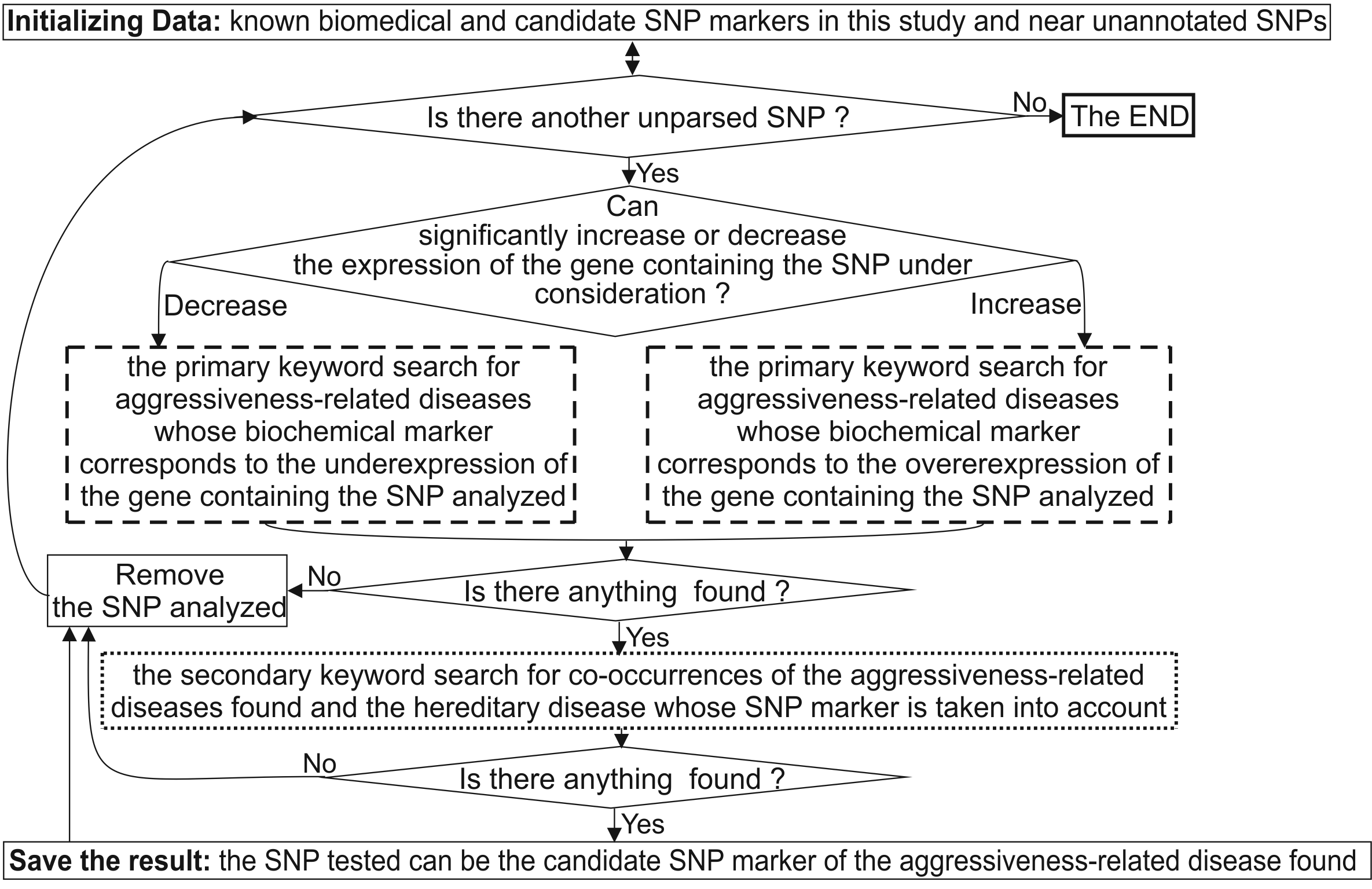

Supplement: Additional file 2: Figure S1. — A flow chart of the keyword search for aggressiveness-related diseases whose biochemical markers correspond to an alteration in expression of the human gene under study containing the candidate SNP marker of interest. Legend: two boxes consisting of a dashed line depict the primary keyword search for diseases whose symptoms include aggressiveness; the box outlined with a dotted line depicts the secondary keyword search for co-occurrence of the aggressiveness-related disease found by the primary keyword search and the hereditary disease clinically associated with the SNP in question. (PNG 385 kb) [file 12864_2016_3353_MOESM2_ESM.png]
